# Characteristics, Opportunities, and Challenges of Osteopathy (COCO) in the Perceptions of Osteopaths in Germany, Austria, and Switzerland: Protocol for a Comprehensive Mixed Methods Study

**DOI:** 10.2196/15399

**Published:** 2019-12-18

**Authors:** Jan Porthun, Jonas Manschel

**Affiliations:** 1 Department of Health Sciences Gjøvik Norwegian University of Science and Technology Gjøvik Norway; 2 Dresden International University Dresden Germany

**Keywords:** osteopath, osteopathic medicine, health occupations, occupational profile, mixed methods

## Abstract

**Background:**

Currently, the importance of osteopathy within the health care system is controversial. The training structures and the acknowledgment of the occupational profile strongly differ in the German-speaking territory.

**Objective:**

This study aims to examine the characteristics of the osteopathic profession as well as the possibilities and challenges for osteopaths in Germany, Austria, and Switzerland.

**Methods:**

This study adopted a mixed methods design. The research topic will be examined based on qualitative and quantitative partial studies that will be conducted in parallel as well as sequentially. By applying different research methods and sample testing and by using standardized, validated measurement methods, we expect to be able to gain new insights into the work area of osteopathy.

**Results:**

In November 2018, we started the research and data collection. Currently, we are conducting the first two partial studies. The planned duration of each of the partial study is 6-9 months. The project is scheduled to be completed in 2021.

**Conclusions:**

This study will examine how osteopaths define themselves in comparison with professionals from other occupational profiles and how they describe the characteristics of their work. The identification of central issues is expected to help clarify the issues and define the profession. As such, the results might contribute to the conservation and improvement of the quality of osteopathic treatment.

**International Registered Report Identifier (IRRID):**

PRR1-10.2196/15399

## Introduction

### Background

The treatment method of osteopathy and the occupational profile of osteopaths have been discussed more often in recent times. However, there is still no uniform international regulation on who is allowed to practice as an osteopath and what qualification he or she requires. Osteopathy has been defined by the European Committee for Standardization as a whole-person, patient-centered, manual health care discipline that emphasizes on the interrelationship of structure and function of the body and facilitates the body’s innate ability to heal itself [1].

An increasing number of European countries, such as Italy and Luxembourg, are developing professional regulations for osteopaths [2]. Thus far, eight countries in Europe (England, Finland, France, Iceland, Lichtenstein, Malta, Portugal, and Switzerland) have legal regulations concerning osteopathy [3].

In Germany, however, *osteopathy* is not an independent profession. Currently, three different professional groups are practicing osteopathy: physicians, natural health professionals, and physiotherapists [4]. However, there is no legal basis for practicing osteopathy and for its rank in health care treatment. Hence, different jurisdictions and the interpretation of osteopathy during the past years have, in practice, resulted in confusion among professionals practicing osteopathy. The Higher Regional Court Düsseldorf, for example, prohibited a physiotherapist from practicing osteopathy at his facility, as long as the treating person had not been appointed as a physician or had received permission to practice healing arts pursuant to § 1 HeilPrG (German law on natural health professionals) [5]. In contrast, in the course of another matter, the Higher Regional Court Frankfurt decided that practicing healing arts will only be reserved for physicians and natural health professionals if the patient’s health is at risk. Such an indirect risk for the patient resulting from an osteopathic intervention could, however, be excluded by order of the treating physician [6].

Austria does not have any current legal regulation for the occupational profile either. Physicians trained in osteopathy and physiotherapists are practicing osteopathy in Austria [7]. During a complete survey in the framework of a master’s thesis, the Austrian *standard osteopath* was described as being female, aged between 30 and 49 years, and primarily qualified as a physiotherapist. Moreover, 77.8% of the interviewees indicated that they did not practice osteopathy exclusively, but mostly in combination with their original profession [8].

In contrast, the term *osteopath* has been protected in Switzerland since 2013 and was acknowledged as a medical profession in 2016 [9].

### Objective

Research concerning the effectiveness of osteopathic treatment methods has advanced lately [10-12]; however, there is only little research about the profession as an osteopath. Therefore, the Characteristics, Opportunities, and Challenges of Osteopathy (COCO) study is supposed to provide clarification on how osteopaths define themselves in comparison with other occupational profiles. The COCO study will examine the characteristics of the osteopathic profession, in addition to the possibilities and challenges, for osteopaths in Germany, Austria, and Switzerland.

Comparable research has already been conducted in other countries. In the framework of the study “Challenges and opportunities for Australian osteopathy: A qualitative study of the perceptions of registered osteopaths,” published in 2018 in the *International Journal of Osteopathic Medicine*, Blaich et al [13] interviewed osteopaths, questioning them about the osteopathic profession. The authors concluded that future osteopathic research and uniform training can strengthen the profession’s position within the health care system. They classified their results as being mostly specific for the Australian context; the results may not transfer to other countries. The qualitative study design is formulated as a potential restriction because the results were acquired from a small sample. With respect to a German-speaking country, this study might be a typical example of a qualitative partial study in the framework of the COCO study. The results of the study by Cerritelli et al [14] provide essential insights into the osteopathic profession in Italy. On the basis of a Web-based survey, the professional profile of an Italian osteopath could be described as that of a freelance, young, and male adult working on his own and having received his training part-time; his original profession is in the fields of sport science or physiotherapy. Concerning the implementation of a license or registration procedure in their country, the authors have pointed out that the different types of professional training must especially be accounted for. This study might be a typical example of a quantitative partial study in the framework of the COCO study.

If we consider both of the indicated studies more closely, the combination of different study designs appears to make sense; therefore, we will be able to examine any data acquired within the intended examination directly with respect to their generality.

The objective of the COCO study is to examine how osteopaths define themselves in comparison with other occupational profiles and how they describe the characteristics of their work.

As the intended research project is very comprehensive, superordinate planning will be required. The objective of this study is to prepare a detailed study design for the more extensive comprehensive study.

## Methods

### Study Design

This study employed a mixed methods design. The research topic will be examined based on qualitative and quantitative partial studies that will be conducted in parallel as well as sequentially.

The parallel form comprises several partial studies during the same period, which will be evaluated subsequently. In contrast, different studies are finally conducted in a sequential form. Thus, evaluation of the qualitative studies will influence the later implementation of the quantitative studies in the planned study: Technically, this is an exploratory design. First, hypotheses can be developed on the grounds of the results from the qualitative study projects, which are then examined in a second step on the generality of the projects. This kind of sequential planning allows for differentiated questioning. In contrast, a parallel design allows for implementation of different study designs without reciprocal influence [15].

### Procedure

Before the study, intensive literature research was conducted. We searched databases for publications referring to the research topic. On the basis of an overview of the current research situation, qualitative partial studies will be implemented as a first step, for example, interviews with osteopaths in Germany, Austria, and Switzerland. Following analytical evaluation of the study contents, questionnaires will be developed with the resulting data. In the second step, the quantitative partial studies will be conducted by standardized questionnaires. These will be filled in by osteopaths working in Germany, Austria, and Switzerland. After conclusion of the survey, meta-integration of the qualitative and quantitative study results will be conducted in a separate partial study. At the end of the integrative work, a quantitative study will be performed to evaluate the entire study project.

### Literature Research

We primarily looked for osteopathic final papers describing the work area of osteopathy in the broadest sense. A search was performed using the internet search engines *Google* and *Google Scholar* as well as via the portals O*steopathicresearch.com*, *Ostemed-dr*, *Pubmed*, *EMBASE*, and *Pedro*. Keywords used for the search were, for example, *Abschlussarbeit Osteopathie*, *Fragebogen Osteopathie*, *Berufsbild Osteopathie*, *dissertation/thesis osteopathy*, *questionnaire osteopathy*, and *occupational profile osteopathy*. In addition, we particularly searched for papers published on the websites of individual training institutions and universities offering osteopathic education.

### Inclusion and Exclusion Criteria of Available Literature

To avoid falsifying the current situation of osteopathy by inclusion of older surveys, only studies published ≤10 years ago have been accounted for. In addition, the full text of the used studies must be available to the authors.

### Inclusion and Exclusion Criteria of Participants

All participants of the individual qualitative and quantitative studies must have attended at least 4 years of training as osteopaths and must be practicing as osteopaths (for humans). Participants are enrolled via the lists of therapists of the professional associations or interest groups. As such, it is guaranteed that the criterion of a comprehensive training in osteopathy will be fulfilled; otherwise, the participants cannot be members of such associations.

Another criterion for the entire group of participants is a balanced gender distribution (as balanced as possible). In addition, participants in the individual countries should be subject to a topographic spread as wide as possible, so that district-specific phenomena can be excluded. In Germany and Austria, osteopathy is not an independent profession; therefore, different professional groups practicing osteopathy in both countries should be represented among the participants of the study.

### Description of the Partial Studies

For the partial studies, each study is conducted by an individual study co-ordinator who has access to the literature, the study protocol, and the datasets resulting from other partial studies.

#### Qualitative Partial Studies—Osteopaths in Germany, Austria, and Switzerland

These partial studies include the planning and conduct of interviews with osteopaths working in Germany, Austria, and Switzerland separately. Subsequently, there will be a qualitative content analysis, according to Mayring [16], to achieve a first overview of the research topic. To acquire representative results, 10-12 interviewees in each country will be questioned per study [16].

#### Quantitative Partial Study—Osteopaths in Germany, Austria, and Switzerland

In the framework of this partial study, a questionnaire survey is planned with closed-ended questions. In Germany, Austria, and Switzerland, there are approximately 10,000, 1000, and 1040 practicing osteopaths, respectively; therefore, we intend to enroll 370, 278, and 281 cases, respectively (95% CI for the respective number of practicing osteopaths), for the questioning to acquire representative results [17-20].

#### Meta-Integration Partial Study

This partial study includes a meta-integration of the results of the individual qualitative and quantitative partial studies.

#### Quantitative Partial Study: Evaluation of the Entire Study Project

In the framework of this partial study, an evaluation of the entire COCO study is planned from a scientific point of view and from the point of view of the participating persons.

### Data Evaluation

#### Phases

In the following paragraphs, individual phases of the described study procedure will be explained in detail. Currently, three evaluation phases are planned:

- Phase 1: Planning and implementation of the qualitative partial studies
- Phase 2: Planning and implementation of the quantitative studies
- Phase 3: Meta-Integration of the different partial studies and evaluation of the project

#### Phase 1: Planning and Implementation of the Qualitative Partial Studies

**Figure 1:**
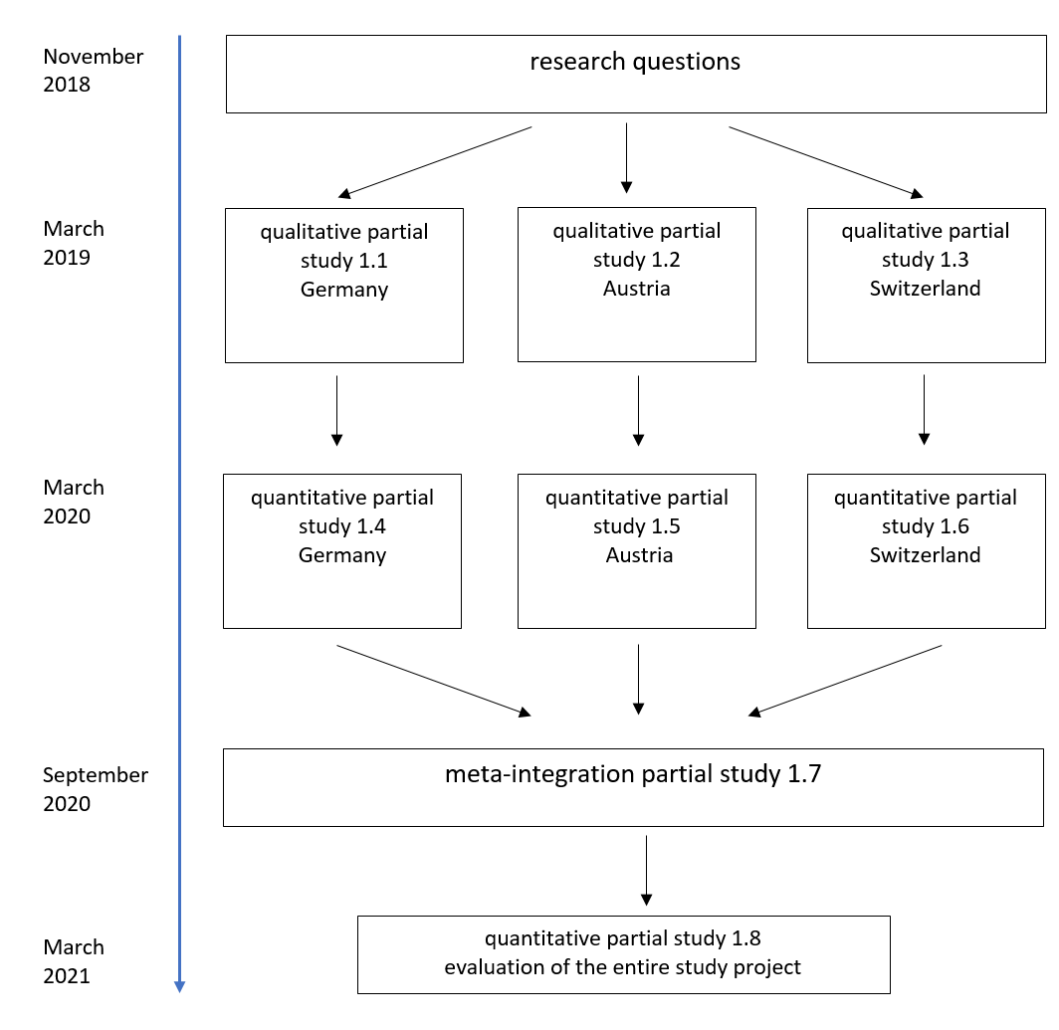
Design of the Characteristics, Opportunities, and Challenges of Osteopathy (COCO) study as a flowchart.

This phase includes reading and evaluation of the available studies to obtain a first overview of the state of research. The studies will be categorized according to the survey period, the type of collected data, and the country of origin. To keep the results as up to date as possible, the studies should be weighted according to their year of publication. For example, studies can be categorized and evaluated according to the periods of 2019-2017, 2016-2013, and 2013-2009, similar to the process used in another mixed methods study by Carsons-Stevens et al [21]. Subsequent to the evaluation of the available literature, an interview guideline will be developed with respect to the research topic. The country-specific questionnaire of participants and the qualitative content analysis are conducted by one study co-ordinator, according to the first three planned partial studies (Figure 1).

The objective is to reduce the material by the subsequent content analysis in such a way that essential content can be filtered out and the research topic can be better understood [22]. A system of categories will be developed to interpret the results of the qualitative partial studies, where the resulting data can be categorized. This system of categories makes the interpretation of the data comprehensible for the readers [22]. On this basis, it will be possible to develop a questionnaire with closed-ended questions in the framework of the following partial studies and to conduct larger surveys.

#### Phase 2: Planning and Implementation of the Quantitative Studies

The results of the partial studies (studies 1.1, 1.2, and 1.3 in Figure 1) will be compiled. The quantitative studies are implemented with the objective of reviewing the results of the qualitative partial studies with respect to the population. By sampling, we will examine how often statements or categories stemming from the qualitative studies occur within the population. As a measuring tool, a standardized questionnaire is developed. The survey will again be implemented in three partial studies that are conducted by one study co-ordinator (studies 1.4, 1.5, and 1.6 in Figure 1). The results will be conveyed in a descriptive manner by preparing schemes in the form of spreadsheets and graphics for the individual items. Before comparison of the individual groups, the data are tested with respect to normal distribution. During the planning of a study, assumptions concerning normal distribution may be based on the results of pilot studies, for example, the studies by Rochon et al and Shuster [23,24]. The visual assessment of a histogram and the Q-Q or P-P plots, as well as the Shapiro-Wilk test, may also be indicated as possible alternatives [25]. The distributions will then be examined with the help of Chi-square and Fisher exact tests. In addition, group comparisons may be conducted using the *t* test or the Wilcoxon-Mann-Whitney test.

#### Phase 3: Meta-Integration of the Different Partial Studies and Evaluation of the Project

The data resulting from the qualitative and quantitative partial studies will be compiled, and the entire study project will be evaluated subsequently. Integration and reflection will be performed after data collection has been completed. It is the objective of the integration to examine which results from the qualitative research can be generalized by the quantitative study by using questionnaires. To compare the data and results, a spreadsheet or concept map will be compiled. As such, it will be possible to evaluate individual items or categories with the respective questions as opposed to entire questionnaires [15].

### Recommendation for Questionnaires

Several aspects must be accounted for with regard to the conception of the measuring instruments. For example, the questions should be short and concrete and formulated in simple words. Hypothetic or leading questions should be avoided [26]. To review the questionnaires developed later with respect to comprehensibility and unambiguity of the questions, as well as concerning the completeness of the possible answers, the execution of a pretest is recommended [27]. The basis of a representative result is also a questionnaire response rate as high as possible. In the case of questionnaire administered in writing, a response rate of 52% (SD 24%) is expected [28]. To increase the motivation of the study’s potential participants, it is possible to offer shopping vouchers or any similar incentive to the study participants.

### Ethical Considerations

Participation in all the studies conducted within this project will be voluntary. Participants will be informed about their right to refuse participation [29]. Participation based on remuneration will also be excluded. For participants of questionnaires administered in writing, this information can be handled, for example, by including the conditions of participation in an annex of the questionnaire, which the participants approve by sending back the survey. All the interview partners will be informed and must have expressed their consent in writing. The anonymity of all the participants will be guaranteed at all times.

## Results

In November 2018, we started the research and data collection. The planned duration of each of the partial studies is 6-9 months. Qualitative study projects are being conducted in parallel and evaluated. Currently, we are running the first two partial studies, 1.1 and 1.2.

Upon conclusion of the qualitative partial studies toward the end of the year 2019, all results will be collected during a period of 2-3 months to plan the following quantitative partial studies. After the development of a questionnaire as a standardized measuring instrument, the provisional start of questioning is planned for March 2020 within the parallel partial studies 1.4, 1.5, and 1.6. Subsequent to the questioning, the research results will be summarized finally in a meta-integration in the form of autonomous study 1.7, which will begin in September 2020. After the expected conclusion of the research project in March 2021, the study will be evaluated with the last partial study 1.8. From the current point of view, the overall results of the project will be published at the end of the year 2021.

## Discussion

This will be the most extensive analysis on the profession of osteopaths in the German-speaking territory. By applying different research methods and sample testing and by using standardized, validated measurement methods, we expect to be able to gain new insights into the work area of osteopathy.

During evaluation of the results, topics concerning the situation of osteopathy in general practice will be identified primarily. The resolution of these central issues is expected to help identify the characteristics of the work area. Delimitation concerning other professional groups will also be possible. Thus, the results may contribute to the conservation and improvement of the quality of osteopathic treatment. Blaich et al [13] highlighted the necessity of advancement in osteopathic education and research through their qualitative study design.

Increased interdisciplinary cooperation between different professional groups can help us achieve a target-oriented and responsible collaboration between osteopaths and other medical professions during the planning and implementation of patients’ treatment. Comparable results were provided by Cerritelli et al [14] who did not only describe the profile of osteopaths practicing in the country but also provided new insights into the cooperation of different medical professions in the health care system [14].

The issue of different professional situations in the countries to be examined should also be mentioned. Currently, there is no legal basis for practicing osteopathy in Germany and Austria, whereas in Switzerland, *osteopathy* is an acknowledged profession. We will observe whether data can be collected to allow for any conclusions on the reasons of the respective professional situations in these countries. Thus, topics primarily concerning improvements in practice and the professional situation may be identified. Notably, different professional situations in the countries surveyed may limit the comparability of the results. Nonetheless, we believe that we will be able to compare the groups to a certain extent because in all three countries, physiotherapists are the ones who mainly practice osteopathy, even if in Germany, many osteopaths additionally have passed an examination as a Heilpraktiker for legal reasons [30-32]. The final results and recommendations will be summarized to be distributed and made available for politics, professional associations, universities, and training institutions. Thus, the results can be used to improve specific and professional education, training, and advanced training possibilities. Tasks and possibilities of the profession will be presented to extend the task profile and increase autonomy.

Owing to the partially parallel design, studies are conducted simultaneously in the framework of the COCO project, particularly in the qualitative research area. These parts may vary with respect to the selection of methods for the questionnaire of participants. Hence, the qualitative content analysis may become potentially more difficult. The chronological order of the individually designed partial studies may also be considered critically. Owing to the sequential design, the evaluation of the results of the qualitative studies must be concluded before the questionnaires can be started. As the different parts of this study need to be coordinated and conducted in a chronological order, the dataset may be falsified between evaluation and review by sampling. Therefore, subsequent studies will be considered to observe any future modifications of the osteopathic profession. The results will consequently also serve as a basis of further development and testing by future research projects. Through a planned questionnaire of all the study co-ordinators, improvement measures may also be revealed within the COCO study.

## Abbreviations

COCO: Characteristics, Opportunities, and Challenges of Osteopathy
